# Application of Artificial Intelligence in Nursing: A Bibliometric Analysis of Global Research Trends

**DOI:** 10.3390/healthcare14040460

**Published:** 2026-02-12

**Authors:** Lorna Kwai Ping Suen, Jing Zhou, Shaolin Chen, Qilian He, Mark Cheuk Man Tsang, Wilson Kin Chung Leung, Simon Ching Lam

**Affiliations:** 1 School of Nursing, Tung Wah College, Hong Kong SAR, China; 2 School of Nursing, Zunyi Medical University, Zunyi 563000, China; 3 Nursing Department, Affiliated Hospital of Zunyi Medical University, Zunyi 563000, China; 4 School of Nursing, Dali University, Dali 671000, China; 5 School of Arts and Humanities, Tung Wah College, Hong Kong SAR, China

**Keywords:** artificial intelligence, clinical decision making, evidence-based practice, healthcare professional, nursing, bibliometric analysis, research hotspots

## Abstract

**Objectives:** With the rapid growth of artificial intelligence (AI) research across journals and conferences, traditional literature reviews face challenges in capturing broad patterns. This bibliometric analysis maps publication trends, geographic and institutional distributions, research themes, and collaboration patterns in AI applications within nursing literature. Using tools such as Bibliometrix, it provides a systematic visualization of these bibliometric features to inform future research directions. **Methods:** Data were retrieved from the Web of Science database (1956–May 2025), yielding 1194 full-text articles. Analyses were performed using CiteSpace, VOSviewer, OriginPro, Pajek, Bibliometrix, and Excel across four domains: (1) publication productivity (yearly output and citations), (2) distribution by country and institution, (3) research hotspots via keyword analysis, and (4) collaborative networks. **Results:** Publications have increased notably since 2012, with 90% of authors contributing only one article. The analysis identifies the 15 most-cited papers, leading journals by output, prominent countries and institutions, and major keyword clusters: (1) AI in nursing education, (2) clinical decision-making and patient care, (3) health informatics and telehealth, and (4) ageing care with robotics. Regression trends indicate rising publication volumes, while network visualizations reveal collaboration patterns. **Conclusions:** This bibliometric analysis maps publication trends, key contributors, and thematic foci in AI applications for nursing. Rising output and collaborations signal growing interest across patient care, education, and informatics. Findings offer a foundation for future interpretive studies on AI integration in nursing practice.

## 1. Introduction

Artificial intelligence (AI) is transforming various sectors, including healthcare, by offering innovative solutions that enhance efficiency, accuracy and patient outcomes. The journey of AI in nursing can be traced back to the early developments in AI technologies in the mid-20th century. The initial focus was on automating routine tasks and supporting decision-making processes [1]. Early applications included rule-based systems that provided diagnostic support and treatment recommendations [2]. In nursing, AI has become a key technology with the potential to transform patient care, administrative tasks and clinical decisions [3,4]. Several reviews have indicated that using machine learning, natural language processing and predictive analytics, AI systems can help nurses manage complex situations, optimise workflows and personalise patient care [5,6]. Moreover, AI technologies offer unprecedented opportunities, enhancing clinical education and training for nurses. Simulated learning environments enable realistic and immersive experiences that prepare nursing professionals for real-world clinical situations [1]. Previous studies on this topic have provided valuable insights into specific aspects of the field; however, they vary in scope and methodology, and a comprehensive bibliometric overview is still limited. Bibliometric studies are a research method that focuses on the quantitative analysis of written publications, such as books and articles [7]. They are particularly prevalent in the field of information science, with some applicable features, including quantitative and citation analysis, co-authorship analysis, and co-word analysis, among others [8,9]. These features enable bibliometric studies to be powerful tools for understanding and evaluating the landscape of scholarly communication and research output, which ultimately develop objective metrics through typical analyses, including trend identification, research impact evaluation, mapping of scientific collaboration, and benchmarking of researchers and institutions. Furthermore, applying Lotka’s law to predict authors’ publication patterns helps to understand the distribution of scientific productivity, identify key contributors, and analyse the dynamics of knowledge production in AI [10].

This bibliometric analysis provides a descriptive and comprehensive overview of the research landscape of AI in nursing. It maps publication trends, research hotspots, and collaborative networks to illustrate how the field has evolved over time. While the study does not assess the quality or practical effectiveness of AI applications, it offers an evidence-based framework to understand current patterns and emerging areas of scholarly attention. These findings can serve as a foundation for future interpretive analyses that explore conceptual, methodological, or policy-related implications in greater depth.

## 2. Methods

### 2.1. Study Design

In our study, we employed a multitool approach to conduct a comprehensive bibliometric analysis, utilising six distinct tools to enhance the depth and validation of our findings. The tools used are as follows: (1) CiteSpace 6.3.R1 Basic (a software tool for calculating the number of articles published in a journal each year), (2) VOSviewer 1.6.20 (a software tool for constructing and visualising bibliometric networks), (3) OriginPro 2025 v10.2 (data analysis and graphing software based on the results generated from VOSviewer), (4) Pajek 6.01 (graphing software based on the results generated from VOSviewer), (5) Bibliometrix (an online analysis platform based on R language, 4.5.0) and (6) Excel (generates figures from exported data). This multitool approach was designed to enhance the analytical perspective and ensure the comprehensiveness, validation and robustness of the findings. Bibliometrics provides a macroscopic view of the research field through publication trends, including productivity reports, distribution reports, research hotspots, keyword usage and cooperative network reports. The Equator’s preliminary guidelines for reporting bibliometric reviews of the biomedical literature (BIBLO) were followed to ensure the transparency and accurate reporting [11].

### 2.2. Data Collection

**Figure 1:**
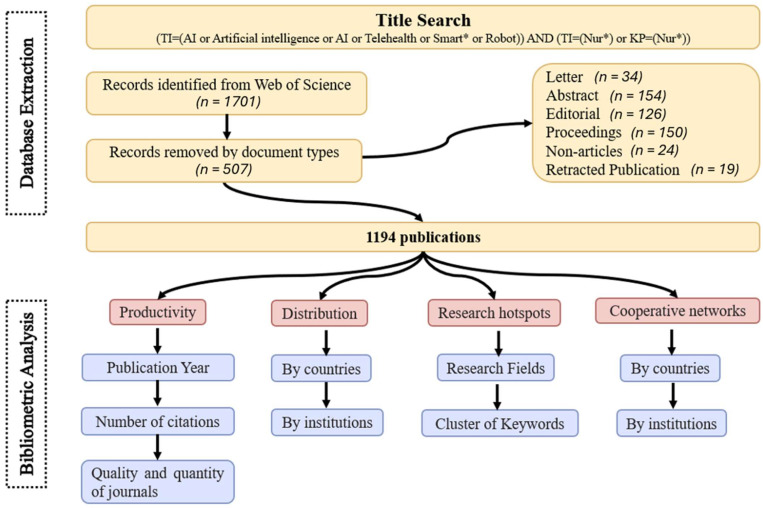
Flow chart of research methodology.

We conducted a topic search, which included searches of the title, abstract and indexing, and used the Web of Science (WOS) core collection database to include articles published from the database’s inception (i.e., January 1956) to May 2025. The search strategy was as follows: (TI = (AI or Artificial intelligence or AI or Telehealth or Smart* or Robot)) AND (TI = (Nur*) or KP = (Nur*)). The inclusion criteria were (1) papers written in English and (2) studies related to nursing using AI technologies. The exclusion criteria were letters, abstracts, editorials, proceedings, nonarticles or retracted publications. The papers’ screening process was conducted by two independent authors (LS and SS), and disagreements between the authors were resolved by discussion. The search identified 1701 publications from the database. After CiteSpace automatically removed 507 ineligible papers, 1194 publications were included in the bibliometric analysis. The flowchart of the research methodology is shown in Figure 1.

### 2.3. Data Analysis

**Figure 2:**
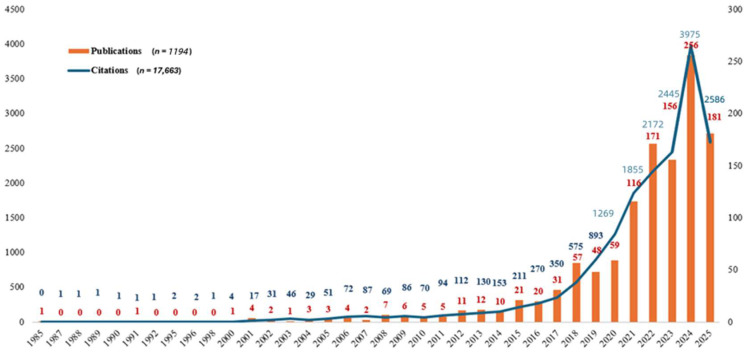
Years of publications and citations (1985–May 2025).

**Figure 3:**
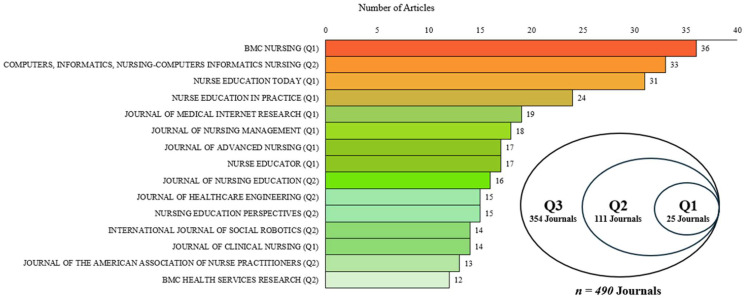
Quality and quantity of journals (1985-May 2025).

**Figure 4:**
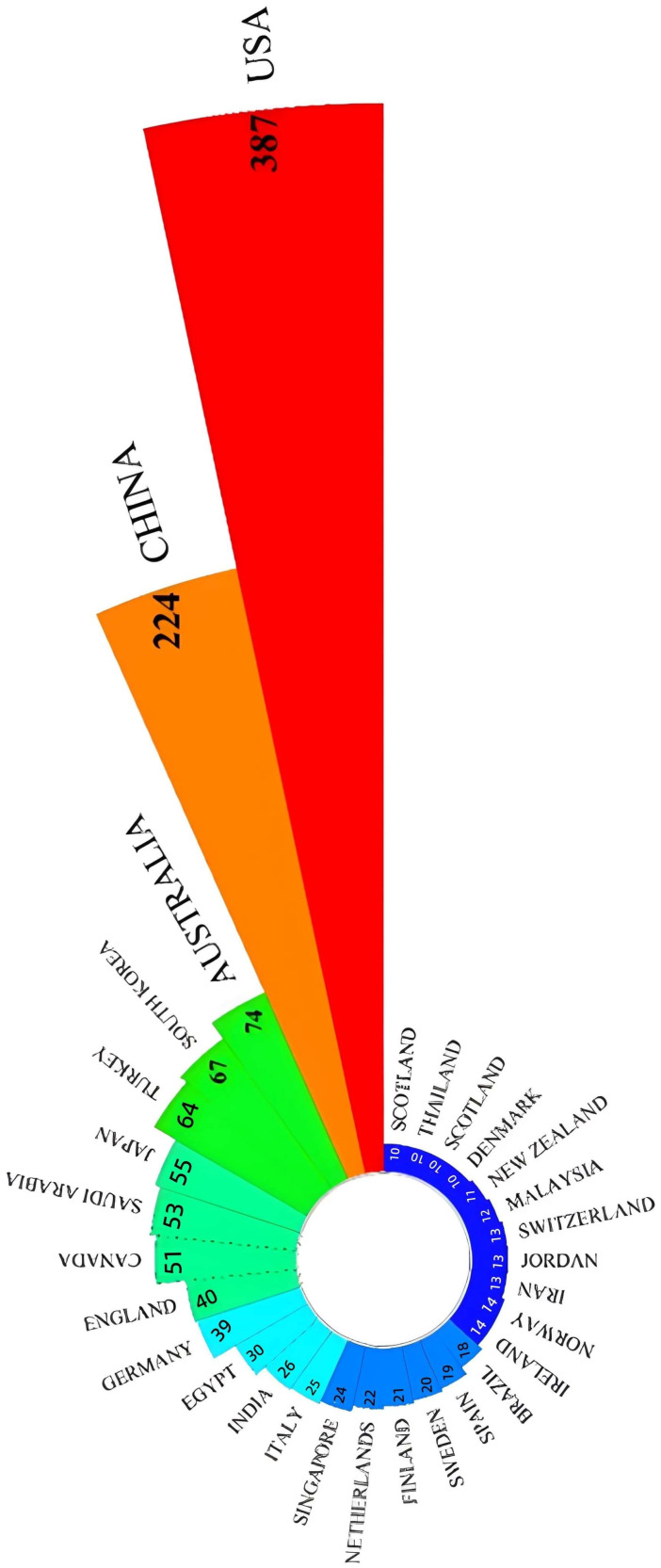
Top 30 countries published AI in nursing.

**Figure 5:**
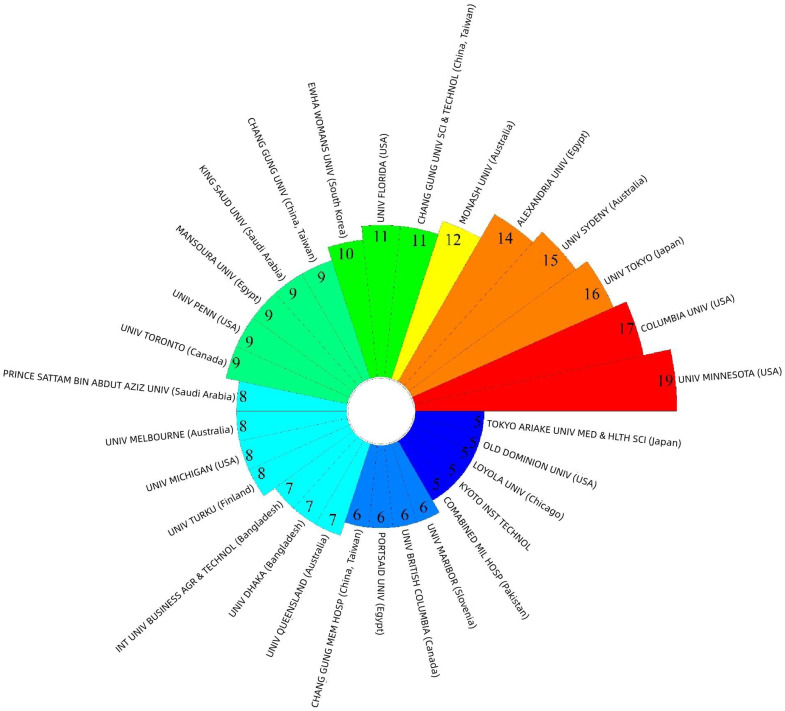
Top 30 institutions published AI in nursing.

The analysis began with the use of CiteSpace 6.3.R1 Basic to examine the publication volume extracted from the WOS database. The annual publication and citation generated for two columns were used to create Figure 2 in Excel. Additionally, Bibliometrix was utilised to analyse journal publication data, resulting in the identification of the 15 journals with the highest number of publications. These findings were used to create Figure 3 in Excel. Afterwards, VOSviewer 1.6.20 was used to analyse publication data by country and institution, exporting relevant names and quantities. These data were further visualised using OriginPro 2025 v10.2 to produce Figure 4 and Figure 5. Moreover, the keyword information and collaboration networks between countries and institutions, as analysed by VOSviewer 1.6.20, were processed with Pajek 6.01 to generate Supplementary Figures S2–S4. The projection of the number of publications and citations was evaluated by regression analyses using SPSS (version 30.0).

## 3. Results

### 3.1. Productivity

As illustrated in Figure 2, research on AI in nursing has evolved since 1985. The integration of AI into nursing has undergone three distinct phases: an initial period characterised by slow development (1977–2003), a gradual upward trend (2004–2017) and a phase of rapid growth (2018–2025). A total of 1194 publications were reported within the study period. A simple linear regression was conducted to examine the relationship between the year and the number of annual publications from 2010 to 2024. The analysis revealed a statistically significant model with R^2^ = 0.756, F (1, 13) = 40.315 and *p* < 0.001. The regression equation was y = −29591.904 + 14.704x, indicating that each additional year was associated with an increase of approximately 14–15 publications. This finding reflects a positive trend in publication output over the past 15 years (Figure 2).

For authorship, there was a notable scarcity of nursing experts specialising in AI, with 90% of scholars having published only one paper. Researchers who have contributed to three or more publications were rare (Supplementary Figure S1). A mathematical formula based on Lotka’s Law was used to describe the productivity of authors in terms of the number of articles they have written. It is a principle in bibliometrics that posits the number of authors publishing a certain number of papers is inversely proportional to the square of the number of documents they have published, and it is used to understand and quantify author productivity in a specific field [10]. On the basis of the observed Lotka’s Law pattern, the proportion of authors who publish multiple articles may continue to increase over the coming years, although this should be interpreted as a descriptive tendency rather than a formal prediction.

In terms of citations, with the advancement of AI technology and the increasing exploration and application of AI in the nursing field, the citation counts have been steadily rising over the years (*n* = 17,663), exhibiting an explosive growth after 2018 in Figure 2. Similar to publications, citations have also presented a positive trend over the years, with a statistically significant model with R^2^ = 0.769, F (1, 13) = 43.257 and *p* < 0.001. The regression equation was y = −457593.350 + 227.35x, implying that each additional year was associated with an upward trend in citations of over 220. Given the historical trend, it is plausible that yearly publications and citations will continue to show an upward trajectory. We present the 15 frequently cited papers since year 2000 in Table 1 [12,13,14,15,16,17,18,19,20,21,22,23,24,25,26], from which the paper published by Rahmani [12] had the highest citation of 644.

Figure 3 illustrates the quality and quantity of journals. A total of 490 core journals have published articles on the domain of AI in nursing (1985 to May 2025). Amongst these journals, 25 were categorised within the Q1 quartile, 111 within the Q2 quartile and 354 within the Q3 quartile. Notably, BMC Nursing, Computers, Informatics, Nursing (CIN) and Nurse Education Today were distinguished as the top three journals commonly publishing articles on AI applications in nursing, with over 30 articles each.

### 3.2. Distribution

Figure 4 displays the number of publications from the top 30 countries, with longer bars indicating a higher number of publications. The United States exhibited the highest productivity (*n* = 387), followed by China (*n* = 224). While a noticeable disparity in publication numbers is observed among countries, this difference may reflect variations in population size, the number of practicing nurses, and the capacity of nursing education and research institutions. Countries with larger nursing workforces and more established research infrastructures, such as the United States, China, and Australia, are therefore more likely to demonstrate higher research productivity. A total of 1875 institutions have contributed literature on AI in nursing. Figure 5 illustrates the publication output of the top 30 institutions. Given that the research and application of AI in nursing are still in their nascent stages, the distribution of institutional contributions is relatively uniform. The University of Minnesota demonstrated the highest productivity (*n* = 19), followed closely by Columbia University (*n* = 17).

### 3.3. Research Hotspots

In bibliometrics, the occurrence of keywords refers to the frequency with which specific terms appear in academic publications, helping identify research trends, map research areas and understand the focus and dynamics of a particular field. We present the density map of the occurrence of keywords related to publications focused on using AI in nursing (Supplementary Figure S2). A cluster refers to a group of related terms that frequently appear together in academic publications, indicating a common theme or topic within the research field. This clustering helps identify major research areas, understand the relationships between different issues and reveal how various concepts are interconnected within the literature. From the keyword clustering analysis by VOSviewer, the current research hotspots are mainly concentrated in four areas, namely, (1) AI-enhanced nursing education, (2) AI-driven clinical decision-making and patient care, (3) health informatics and telehealth and (4) AI-driven healthcare for ageing and robotics. Examples of these typical research hotspots are illustrated in Table 2.

### 3.4. Cooperative Networks

Supplementary Figure S3 illustrates the collaborative relationships between different countries, where the connecting lines represent collaborations, and the thickness of the lines indicates the strength of these collaborations. A noteworthy aspect is the strong partnerships that the United States and China have established with various countries in the field of AI in nursing, driven by complementary AI strengths. Shared healthcare challenges, academic collaborations, strategic leadership goals and the need for coordinated responses to health crises also influence these partnerships [41]. For institutions, we analysed the collaboration of various institutions to reveal their publication patterns and trends. A total of 1875 institutions have contributed literature on AI in nursing. The frequency of collaboration appears relatively uniform across different institutions (Supplementary Figure S4). The checklist for reporting bibliometric reviews of the biomedical literature (BIBLIO) published by the EQUATOR network is displayed in Supplementary Figure S5.

## 4. Discussion

### 4.1. Strengths of Using Bibliometric Analysis in This Study

Bibliometric analysis offers numerous strengths and advantages in the study of AI in nursing. This bibliometric analysis offers a thorough examination of the research landscape surrounding artificial intelligence (AI) in nursing. It provides valuable insights into prevailing trends and a comprehensive overview of the AI research landscape in nursing, offering insights into the current trends, existing challenges, and prospective developments within the field.

By exploring global research patterns and identifying key areas of focus, this study equips researchers, policymakers, and practitioners with the knowledge needed to navigate the dynamic evolution of AI in nursing. Such understanding fosters continued innovation and supports the ethical advancement of technologies that could inform nursing practice and improve patient outcomes.

### 4.2. Changing Publication Patterns on AI in Recent Years and Their Impact

The publication patterns in AI have undergone significant changes in recent years, with a substantial increase in the number of publications. A total of 79 countries have published literature on AI in nursing, showing global interest in the field. This surge reflects the growing interest and advances in AI technologies. Moreover, the increased volume of research has accelerated the dissemination of knowledge and the pace at which discoveries are applied in practice. As a result, this rapid growth in AI research has become an integral part of nursing practice, providing tools that enhance patient care, streamline workflows and support informed clinical decision-making. For example, by analysing large datasets, AI can help identify patients at risk of complications, enabling positive interventions [42]; assist nurses in making informed decisions and improve patient safety and care quality [43]; or AI-driven tools are being used to automate and improve the accuracy of nursing documentation, reducing the administrative burden on nurses and offering additional time for direct patient care [44].

### 4.3. Journal Quartile Distribution of AI Nursing Publications

The quartile ranking of journals (Scopus SJR 2024) provides a proxy indicator of journal prestige based on citation impact. As shown in Figure 3, these rankings reveal that articles on AI in nursing appeared across 490 journals from 1985 to May 2025. Notably, BMC Nursing and CIN ranked as leading outlets, publishing 36 and 33 articles, respectively, on AI applications in nursing. While some publications appeared in higher-quartile journals (Q1 and Q2), substantial dissemination occurred in lower-quartile journals (Q3 and Q4). Future research could prioritize higher-quartile journals (Q1/Q2) to potentially increase visibility and citation impact within the field.

### 4.4. Trend in the Number of Citations on Papers Using AI in Recent Decades

The trend in the number of citations on AI-related papers has seen a significant increase in recent decades. This trend can be attributed to several factors. Firstly, the rapid advancements in AI technology have led to numerous practical applications of AI in various fields, including nursing. As these technologies prove their utility and effectiveness, the foundational research supporting them becomes valuable and thus frequently cited. Secondly, the interdisciplinary nature of AI research means that it often intersects with multiple fields, such as engineering [45], health informatics [38], psychology [46] and public health [47], leading to a broad audience and great citation potential. These examples illustrate the collaborative efforts across disciplines to leverage AI’s potential in nursing, ultimately contributing to enhanced patient care and improved health outcomes. The explosive growth in citations after 2020 suggests a tipping point where AI research in nursing has moved from being a niche area of interest to a mainstream topic of significant importance. This movement can be seen as a validation of the field’s maturity and its critical role in advancing healthcare practices. The rising citation counts highlight the importance of staying current with the latest research. It also underscores the significance of contributing high-quality research that can withstand the test of time and remain relevant as the field continues to evolve.

### 4.5. Lotka’s Law and the Prediction of Authors’ Publications on AI

Lotka’s Law, first proposed by Alfred J. Lotka in 1926, describes how author productivity within a research field typically follows an inverse distribution—where a small number of researchers produce the majority of publications, while most contribute only once [11]. In the present analysis, approximately 90% of authors in AI and nursing have published a single paper, with fewer than 10% producing three or more. This pattern indicates that the field remains in an early developmental phase. Advancing research in this area demands dual expertise in nursing and computational science—skills that are still limited and require extensive training. Moreover, the rapid evolution of AI technologies challenges researchers to continually update their knowledge, which may constrain sustained publication activity. As the field matures, its publication distribution may gradually converge with classical Lotka’s Law trends, characterised by the emergence of a core group of prolific authors. Strengthening research capacity through targeted funding, interdisciplinary collaboration, and specialised training could encourage continuity and deepen expertise in AI-focused nursing research.

### 4.6. Expansion of Cooperative Networks

The growth of cooperative networks has enhanced both interdisciplinary and cross-national collaboration in AI-related nursing research, contributing to a more dynamic exchange of knowledge, expertise, and methodological approaches. The analysis revealed that Saudi Arabia and Egypt exhibited the most extensive collaborative links, followed by China and Japan. At the institutional level, Chang Gung University and Chang Gung University of Science and Technology in Taiwan showed strong partnership patterns, while the International University of Business Agriculture and Technology and the University of Dhaka in Bangladesh were also among the most connected institutions. The predominance of countries such as the United States and China in AI collaboration networks likely stems from their robust research capacity, substantial funding for AI and healthcare innovation, and the presence of established international research alliances. The widespread use of English in scientific publishing further supports their global reach and visibility. Conversely, limited representation from many Global South nations may be linked to gaps in research investment, technological infrastructure, and opportunities for international engagement. Promoting equitable partnerships, training, and resource sharing could help address these disparities, fostering broader participation and sustainable progress in AI-focused nursing research. Strengthened global collaboration remains essential for enhancing diversity of perspectives and advancing responsible AI integration into healthcare practice [41].

### 4.7. Different Research Hotspots Related to the Use of AI in Nursing

The application of AI in nursing research has a wide range of implications across various domains, including nursing education, clinical decision-making, telehealth, nursing informatics and care for older adults. AI-enhanced nursing education could promise to transform how future nurses are trained via the integration of AI in nursing simulation education [27,28,29]; AI-driven clinical decision-making and patient management can improve patient outcomes through personalised care at different settings [29,30,31,32,33,34,35]; the integration of health informatics and telehealth expands access to care, especially in remote areas [36,37,38]. Additionally, AI-driven healthcare for ageing populations is becoming increasingly significant, particularly through the use of social robots to reduce depression and agitation [20,39] or by employing machine learning for fall prevention [40]. The growing body of AI-focused nursing research highlights themes of efficiency, personalisation, and improved outcomes as key areas of scholarly exploration. However, many of these studies also raise challenges, such as ensuring data privacy, addressing ethical considerations and providing nurses with adequate training to use AI technologies effectively. Future directions should aim to establish ethical guidelines, improve interoperability standards and promote interdisciplinary collaboration, thereby optimising AI’s benefits in the nursing field.

### 4.8. Limitations and Recommendations

Bibliometric analysis is a valuable method for examining trends and patterns in research, but it has several limitations when applied to studying the application of AI in nursing. This kind of analysis often emphasises quantitative metrics, such as citation counts, which may not accurately reflect the quality or impact of the research. Moreover, AI is rapidly evolving, and bibliometric analysis may not capture the latest developments of emerging trends. Therefore, future studies may consider combining bibliometric analysis research methods with qualitative assessments to evaluate the significance and quality of research, possibly through expert reviews or content analysis, and to assess the direct impact of AI applications on patient care and outcomes, thereby validating the benefits claimed by technological advancements. Moreover, the exclusive use of the Web of Science database may introduce selection bias, potentially resulting in an incomplete representation of the broader research landscape.

In this study, the search strategy combined both targeted and broader keywords—such as “artificial intelligence,” “smart,” and “telehealth”—using the Boolean operator “OR” to capture a wide range of potentially relevant publications. While this comprehensive approach may have included some papers not exclusively centered on artificial intelligence, it was intentionally designed to account for the evolving and often overlapping vocabulary within digital health research, where AI is frequently integrated into broader contexts like smart health or telehealth systems. To minimize the risk of overinclusion, strict AI-specific eligibility criteria were applied during the screening and selection process, ensuring that only studies explicitly involving AI were analyzed. This strategy sought to achieve an optimal balance between breadth and precision while upholding methodological rigor.

By leveraging the strengths of bibliometric analysis and addressing its limitations, the research community can continue to advance the field, ensuring responsible and impactful developments in AI technologies that enhance nursing practice and improve patient outcomes.

## 5. Conclusions

This bibliometric analysis provides a descriptive overview of the global research landscape on artificial intelligence (AI) in nursing, mapping publication trends, key contributors, and thematic developments. The increasing research activity on AI applications in nursing highlights scholarly exploration of its potential across multiple domains, including patient care and operational efficiencies. The rapid growth in research output and international collaborations reflects growing global interest in AI’s role in healthcare. These findings underscore the expanding scope and interdisciplinary nature of this field. Ongoing efforts in these areas may support more effective integration of AI technologies into nursing contexts. By presenting an evidence-based overview of existing research patterns, this study offers a foundation for future investigations to conduct more interpretive, theoretical, or practice-oriented analyses that further clarify AI’s contributions to nursing and healthcare outcomes.

## Figures and Tables

**Table 1:** Top 15 Most cited articles in recent years (1985–2025).

No.	Paper	First Author & Year	Number of Citations	Study Design	Key Findings
1	Exploiting smart e-Health gateways at the edge of healthcare Internet-of-Things: A fog computing approach	Rahmani 2018 [12]	644	Review	The concept design illustrates an IoT-based health monitoring system that enhances system intelligence, energy efficiency, mobility, performance, interoperability, security, and reliability.
2	Acceptance of healthcare robots for the older population: Review and future directions	Broadbent 2009 [13]	477	Review	The review emphasises the importance of incorporating insights from diverse stakeholders, including older adults, their families, and healthcare professionals, and thoroughly assessing their expectations and needs.
3	Efficacy of nurse telehealth care and peer support in augmenting treatment of depression in primary care	Hunkeler 2000 [14]	373	RCT	Telehealth services provided by nurses lead to better clinical outcomes for antidepressant treatments and higher patient satisfaction, making them well-suited for busy primary care environments.
4	The psychosocial effects of a companion robot: A randomized controlled trial	Robinson 2013 [15]	328	RCT	The robotic companion Paro offers positive contributions to nursing home care for older adults. It can potentially meet specific needs that live animals might not fulfill, especially in combating loneliness.
5	Socially assistive robots in elderly care: A mixed-method systematic literature review	Kachouie 2014 [16]	250	Systematic review	This review suggests that robots can significantly enhance the well-being of elderly individuals while reducing the workload of nurses. Moreover, robots that enhance multiple aspects of elderly well-being tend to be more widely accepted.
6	Service robots: Value co-creation and co-destruction in elderly care networks	Caic 2018 [17]	247	Qualitative Research	Elderly individuals seek to achieve the following within their value networks: 1. physical health, 2. psychosocial health, and 3. cognitive health.
7	Effects of robot-assisted activity for elderly people and nurses at a day service center	Wada 2004 [18]	197	Survey	Interaction with robots led to improved emotional states among elderly individuals. Urinary tests indicated that their stress management abilities were also enhanced. Furthermore, the nursing staff experienced reduced stress levels since the elderly needed less supervision during robot interactions.
8	A survey of robots in healthcare	Kyrarini 2021 [19]	193	Review	The paper offers comprehensive insights into cutting-edge research on robots used for care, hospital assistance, rehabilitation, and walking aids, while addressing the challenges healthcare robots face in societal integration.
9	Effects on symptoms of agitation and depression in persons with dementia participating in robot-assisted activity: A cluster-randomized controlled trial	Jøranson 2015 [20]	168	RCT	Robot Paro could be an effective nonpharmacological option for treating neuropsychiatric symptoms and should be viewed as a valuable tool in clinical settings.
10	Competencies required for nursing telehealth activities: A delphi-study	van Houwelingen 2016 [21]	159	Survey	The nursing telehealth entrustable professional activities (NT-EPAs) and associated competencies outlined in this study may serve as a resource for nursing schools aiming to incorporate or expand telehealth education in their curricula.
11	Home telehealth for diabetes management: a systematic review and meta-analysis	Polisena 2009 [22]	158	Systematic review	Home telehealth helps reduce the number of hospital admissions, hospitalisation durations, and bed usage. Across studies, home telehealth was comparable or superior to traditional care in terms of quality-of-life and patient satisfaction metrics.
12	Telehealth and eHealth in nurse practitioner training: Current perspectives	Rutledge 2017 [23]	134	Review	Training through a multimodal approach enabled students to acquire the comfort, knowledge, and skills necessary to adopt telehealth in healthcare settings.
13	Artificial intelligence in nursing: Priorities and opportunities from an international invitational think-tank of the Nursing and Artificial Intelligence Leadership Collaborative	Ronquillo 2021 [24]	130	Review	AI technologies can be valuable tools in empowering nurses to contribute to advancing the nursing profession and enhancing population and global health outcomes.
14	Nursing professionals’ experiences of the facilitators and barriers to the use of telehealth applications: A systematic review of qualitative studies	Koivunen 2018 [25]	130	Systematic review	The facilitators and obstacles were classified into five primary categories, encompassing nurses’ skills and attitudes, their work and operational practices, organisational elements, patient-related factors, and technology.
15	Effects of telehealth by allied health professionals and nurses in rural and remote areas: A systematic review and meta-analysis	Speyer 2018 [26]	127	Systematic review	Telehealth services can potentially match the effectiveness of in-person interventions, offering promising advantages in terms of improved healthcare access, time efficiency, and cost-effectiveness, particularly in rural and remote regions.

**Table 2:** Examples of common research hotspots using AI in nursing.

Research Hotspots	Paper	First Author & Year	Study Type	Key Findings
AI-enhanced nursing education	How artificial intelligence (AI) supports nursing education: profiling the roles, applications, and trends of AI in nursing education research (1993–2020)	Hwang 2024 [27]	Systematic review	A thorough analysis of 112 studies on AI-supported nursing education showed that AI is primarily used for profiling and predicting outcomes in nursing research (63%), with intelligent agents being the most prevalent AI system in this field (53%). The majority of studies employed a quantitative methodology (87%), focusing heavily on health and medical topics (92%).
	The role of artificial intelligence in shaping nursing education: A comprehensive systematic review	Ma 2025 [28]	Systematic review	A systematic review encompassing 15 studies demonstrated that artificial intelligence benefits students in three main areas: attitudes and psychological responses towards learning, learning efficiency, and overall nursing clinical skills.
	Integration of artificial intelligence in nursing simulation education	Chan 2025 [29]	Scoping review	An examination of 14 articles identified AI’s role in prebriefing using chatbots (*n* = 2), simulation through virtual environments (*n* = 11), and debriefing via feedback (*n* = 1).
AI-driven clinical decision making and patient care	Artificial intelligence in nursing and midwifery: A systematic review.	O’Connor 2023 [30]	Systematic review	Digital health data should be established to facilitate the testing, application, and evaluation of AI in nursing and midwifery. Educational programs must be developed to teach these professions about AI, empowering them to lead and participate in digital healthcare initiatives.
	Effect of an artificial intelligence decision support tool on palliative care referral in hospitalized patients: A randomized clinical trial.	Wilson 2023 [31]	Randomized clinical trial	The research employed a pragmatic clinical trial with a cluster-randomised, stepped-wedge approach across 12 nursing units in two hospitals over 15 months. Out of 2544 patients, 1212 (48%) were assigned to use the decision support tool, while 1332 (52%) received standard care. The integration of an AI/ML-powered decision support tool into palliative care practice showed a rise in consultation rates for palliative care among hospitalized patients and contributed to a decrease in hospitalization rates.
	Navigating ethical considerations in the use of artificial intelligence for patient care: A systematic review.	Badawy 2024 [32]	Systematic review	While AI presents significant advantages for nursing practice, it also poses ethical challenges that need careful consideration. To ethically integrate AI into nursing, it is crucial to enhance nursing education, engage stakeholders, and establish comprehensive policies.
	The application and use of artificial intelligence in cancer nursing: A systematic review.	O’Connor 2024 [33]	Systematic review	AI has been utilized across various medical fields, including care for breast, colorectal, liver, and ovarian cancers, among others.
	Artificial intelligence in critical care nursing: A scoping review	Park 2025 [34]	Scoping review	AI-driven documentation tools have proven effective in reducing administrative workload and increasing accuracy, while resource allocation systems have optimized staffing and workflow management in intensive care settings.
	The effects of applying artificial intelligence to triage in the emergency department: A systematic review of prospective studies.	Yi 2025 [35]	Systematic review of prospective studies	Emergency department triage nurses can employ AI as a supportive tool for triage, aiming to reduce undertriage and enhance patient outcomes.
Health informatics and telehealth	Artificial intelligence-assisted telehealth for nursing: A scoping review.	Choi 2023 [36]	Scoping review	AI-assisted telehealth interventions have shown efficiency and promise, suggesting they could be an effective method for delivering care in nursing.
	Specialized nurses’ role in ensuring patient safety within the context of telehealth in home care: A scoping review.	Vaismoradi 2024 [37]	Scoping review	The analysis highlighted the vital contribution of specialised nurses in utilising telehealth within healthcare to achieve top-tier care standards, fostering an atmosphere that emphasises patient safety and well-being in home care settings.
	The evolving role of nursing informatics in the era of artificial intelligence	Nashwan 2025 [38]	Narrative review	AI presents promising advancements in nursing informatics, fostering more efficient patient care and enhanced decision-making. However, addressing ethical issues and ensuring nurses are literate in AI are essential for its successful implementation.
AI-driven healthcare for aging and robotics	Effects on symptoms of agitation and depression in persons with dementia participating in robot-assisted activity: A cluster-randomized controlled trial.	Jøranson 2015 [20]	Cluster-randomized controlled trial	The study involved sixty residents from adapted nursing home units who were diagnosed with dementia or had cognitive impairments. It demonstrated that using Paro (a baby harp seal, which is an adaptive robot with artificial intelligence software) in activity groups for seniors with dementia in nursing homes had a lasting impact on reducing depression and agitation. Paro may be an effective non-pharmacological option for addressing neuropsychiatric symptoms and could be considered a valuable tool in clinical settings.
	Nursing and rehabilitative care of the elderly using humanoid robots	Tanioka 2019 [39]	Review	Previous studies exploring the dynamics of relationships between humans and robots equipped with artificial super-intelligence were analyzed and discussed. This was done to help develop suitable programs with intelligent machines to address the future needs of healthcare. Collaborative efforts involving diverse interdisciplinary activities between human nurses and humanoid nursing robots are envisioned as a promising approach, particularly inspired by Japanese concepts of human caring for an aging population.
	Artificial intelligence for falls management in older adult care: A scoping review of nurses’ role.	O’Connor 2022 [40]	Scoping review	Evidence demonstrates that numerous AI techniques, especially machine learning, are employed to identify risk factors for falls and develop predictive models aimed at preventing falls among older adults, with nurses actively leading and engaging in this research.

## Data Availability

No new data were created or analyzed in this study. Data sharing is not applicable to this article.

## Supplementary Materials

The following supporting information can be downloaded at: https://www.mdpi.com/article/10.3390/healthcare14040460/s1, Figure S1: Author’s publications and prediction; Figure S2: Density map of keywords; Figure S3: Top 30 cooperative networks by countries; Figure S4: Top 30 cooperative networks by institutions; Figure S5: The BIBLIO checklist for reporting the bibliometric reviews of the biomedical literature; Table S1: The use of software for conducting bibliometric analyses.
