# Supplementary material for: Application of Artificial Intelligence in Nursing: A Bibliometric Analysis of Global Research Trends
Source: Healthcare (Basel). 2026 Feb 12;14(4):460. doi: 10.3390/healthcare14040460 (PMC12940271; doi:10.3390/healthcare14040460)
Supplement: Supplementary file 1 [file healthcare-14-00460-s001.zip › Supplementary Table 1.pdf]

Supplementary Table S1. The use of software for conducting bibliometric analyses.

Result		Software
Figures or Tables	Figure 1. Flow chart of research methodology	Powerpoint
	Figure 2. Years of publication and citations	Excel, Citespace
	Figure 3. Quality and quantity of journals (1985-2025)	Bibliometrix (Under R), Excel
	Figure 4. Top 30 countries published AI in nursing	VOSviewer, OriginPro,
	Figure 5. Top 30 institutions published AI in nursing	VOSviewer, OriginPro,
Supplementary files	Figure S1 Author's Publications and Prediction	Bibliometrix (Under R), Excel
	Figure S2 Density Map of Keywords	VOSviewer, Pajek
	Figure S3 Top 30 cooperative networks by countries	VOSviewer, Pajek
	Figure S4 Top 30 cooperative networks by institutions	VOSviewer, Pajek

Note : (1) VOSviewer (a software tool for constructing and visualising bibliometric networks); (2) OriginPro (data analysis and graphing software based on results generated from VOSviewer); (3) Bibliometrix (an online analysis platform based on R language); and (4) Pajek (a graphing software based on results generated from VOSviewer)
