# Supplementary figures and images for: Application of Artificial Intelligence in Nursing: A Bibliometric Analysis of Global Research Trends
Source: Healthcare (Basel). 2026 Feb 12;14(4):460. doi: 10.3390/healthcare14040460 (PMC12940271; doi:10.3390/healthcare14040460)

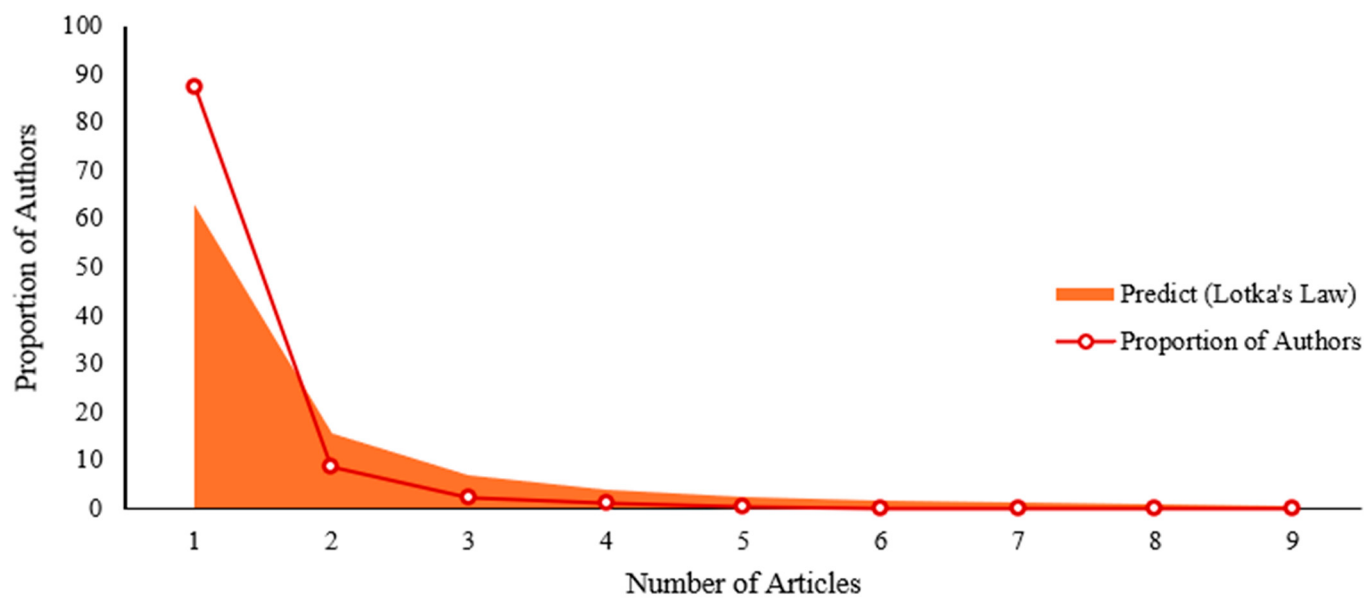

Supplementary Figure S1 Author's publications and prediction

Supplement: Supplementary file 1 [file healthcare-14-00460-s001.zip › Supplementary Figure S1.pdf]

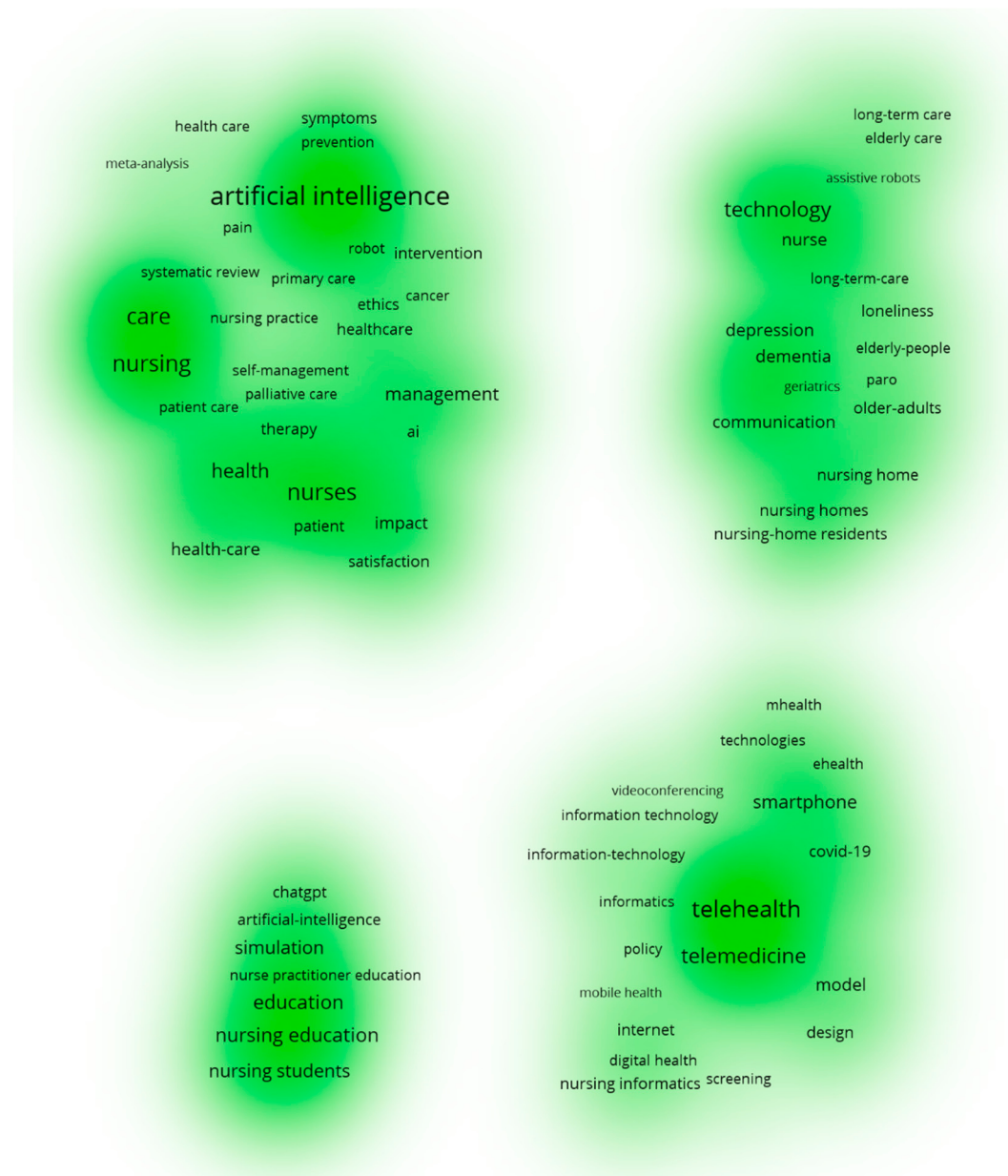

Supplementary Figure S2. Density map of keywords

Supplement: Supplementary file 1 [file healthcare-14-00460-s001.zip › Supplementary Figure S2.pdf]

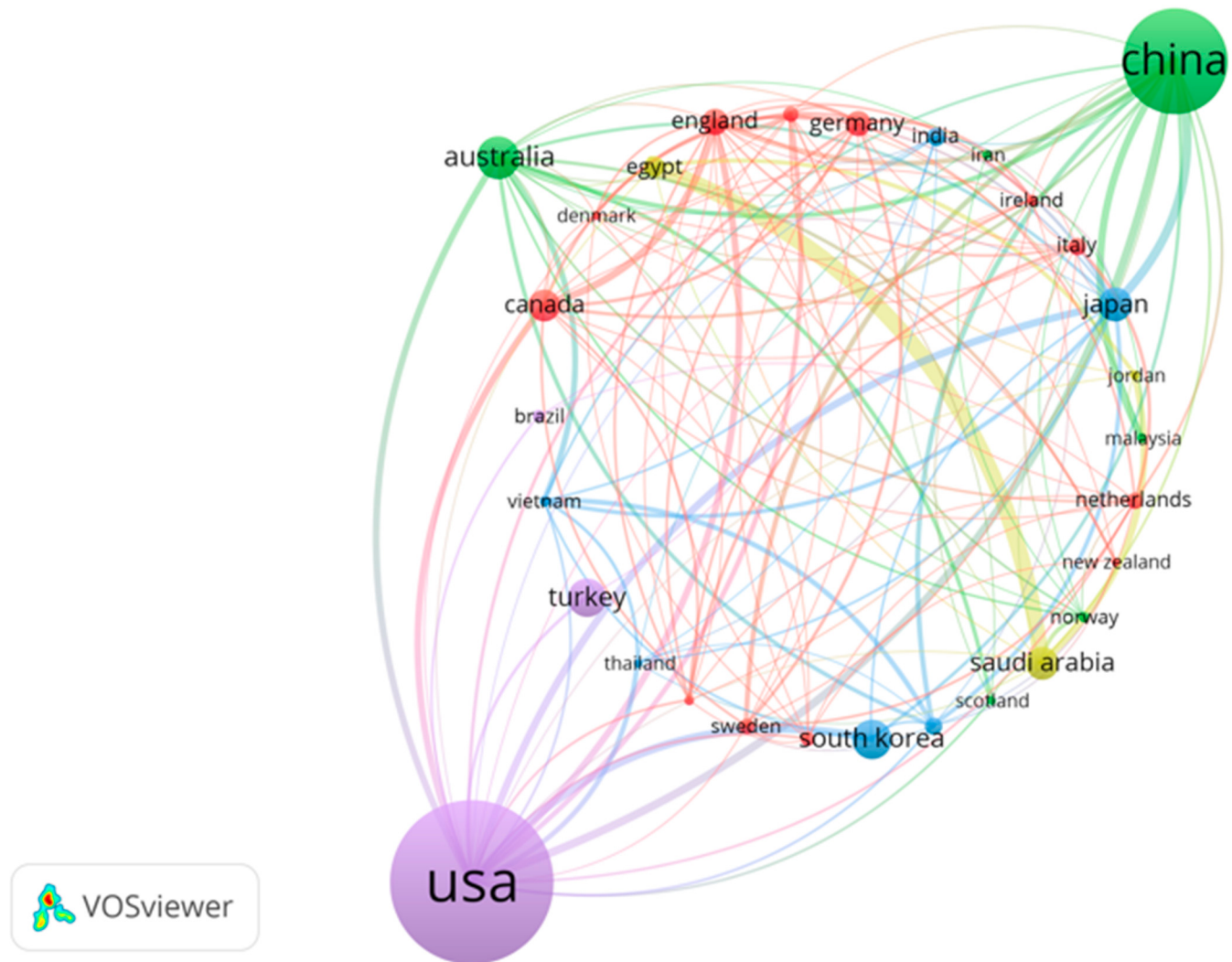

Supplementary Figure S3. Top 30 cooperative networks by countries

Supplement: Supplementary file 1 [file healthcare-14-00460-s001.zip › Supplementary Figure S3.pdf]

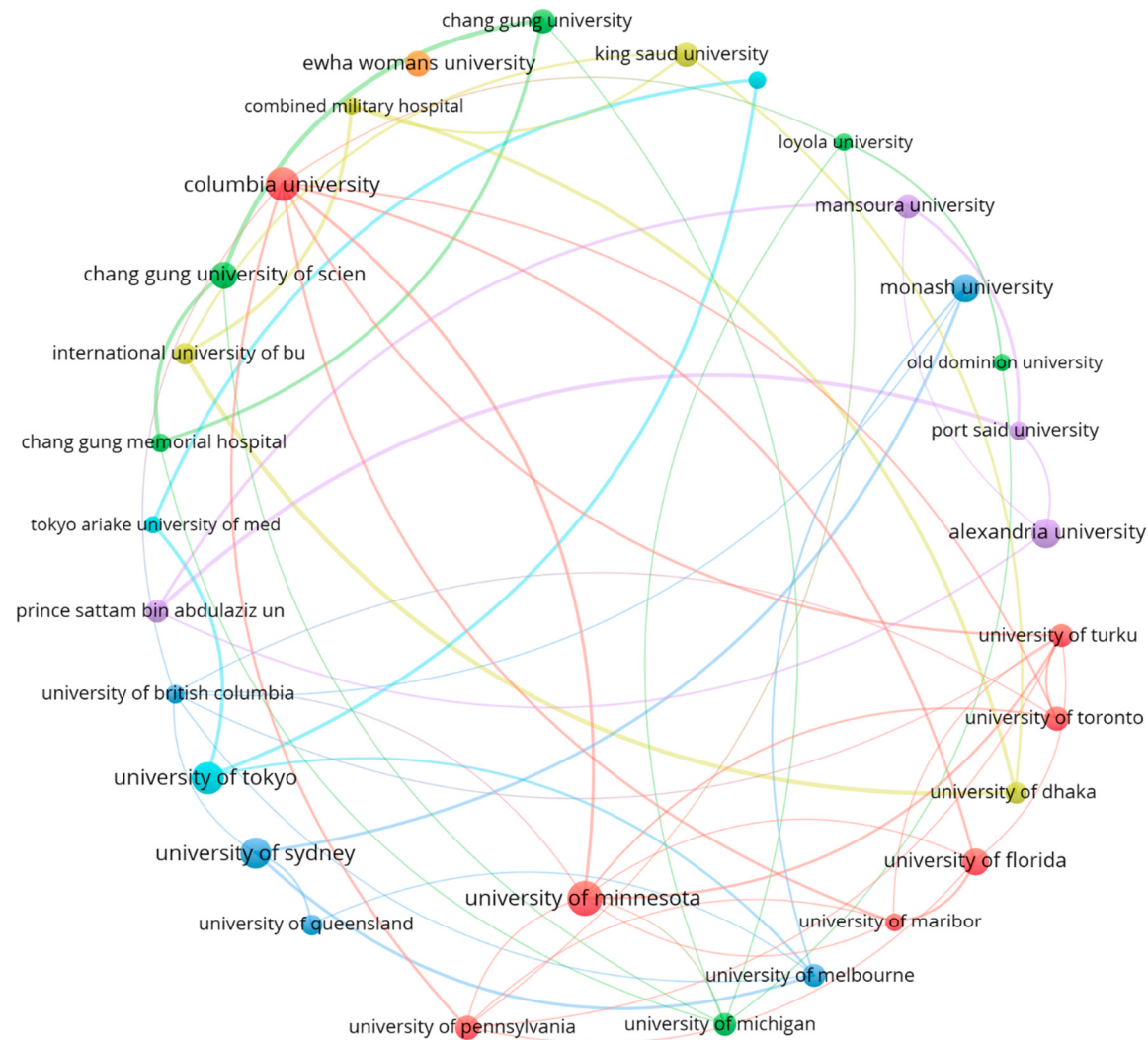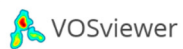

Supplementary Figure S4. Top 30 cooperative networks by institutions

Supplement: Supplementary file 1 [file healthcare-14-00460-s001.zip › Supplementary Figure S4.pdf]
